# Holistic bioengineering: rewiring central metabolism for enhanced bioproduction

**DOI:** 10.1042/BCJ20170377

**Published:** 2017-11-16

**Authors:** Selçuk Aslan, Elad Noor, Arren Bar-Even

**Affiliations:** 1 Max Planck Institute of Molecular Plant Physiology, Am Mühlenberg 1, Potsdam-Golm 14476, Germany; 2 Institute of Molecular Systems Biology, ETH Zurich, Auguste-Piccard-Hof 1, Zürich 8093, Switzerland

**Keywords:** anaplerosis, central metabolism, glycolysis, metabolic engineering, NADPH production, TCA cycle

## Abstract

What does it take to convert a living organism into a truly productive biofactory? Apart from optimizing biosynthesis pathways as standalone units, a successful bioengineering approach must bend the endogenous metabolic network of the host, and especially its central metabolism, to support the bioproduction process. In practice, this usually involves three complementary strategies which include tuning-down or abolishing competing metabolic pathways, increasing the availability of precursors of the desired biosynthesis pathway, and ensuring high availability of energetic resources such as ATP and NADPH. In this review, we explore these strategies, focusing on key metabolic pathways and processes, such as glycolysis, anaplerosis, the TCA (tricarboxylic acid) cycle, and NADPH production. We show that only a holistic approach for bioengineering — considering the metabolic network of the host organism as a whole, rather than focusing on the production pathway alone — can truly mold microorganisms into efficient biofactories.

## Introduction

The bioproduction of value-added chemicals is gaining momentum, slowly but surely replacing environmentally unsustainable fossil-carbon-based chemical processes. Metabolic engineering of microbes now supports the production of food additives [1], plastic monomers [2] and polymers [3], solvents [4], aromatics [5], pharmaceuticals [6], pigments [7], hydrocarbons [8], fuels [9], and chemical building blocks [10]. Successful engineering of a microbe for the efficient bioproduction of a compound requires more than just the introduction of the biosynthesis enzymes. For instance, adaptation of foreign genes into the host organism, in terms of GC content or codon utilization, plays an important role in obtaining high protein expression efficiency and enzymatic activity [11]. Fine-tuning of enzyme levels using different plasmid backbones [12], promoters [13], and ribosome-binding sites [14] is also vital to ensure sufficient flux via the biosynthetic route, while minimizing protein burden and accumulation of toxic or wasteful intermediates [14].

Yet, apart from the properties of the bioproduction pathway itself, as a standalone unit, the endogenous metabolic network of the host organism plays a key role in determining biosynthesis efficiency. There are three primary means by which central metabolism can be modified to enhance a bioproduction process: decreasing and abolishing metabolic flux toward competing pathways that lead to waste byproducts, increasing the availability of the direct precursors of the desired biosynthesis routes, and ensuring high availability of cellular energetic resources, mainly ATP and NADPH. These metabolic strategies are the focus of this review. We argue that engineering of the cell as a whole is crucial for the optimization of any metabolic process. We find the term holistic bioengineering most appropriate to describe this approach.

**Figure 1.**
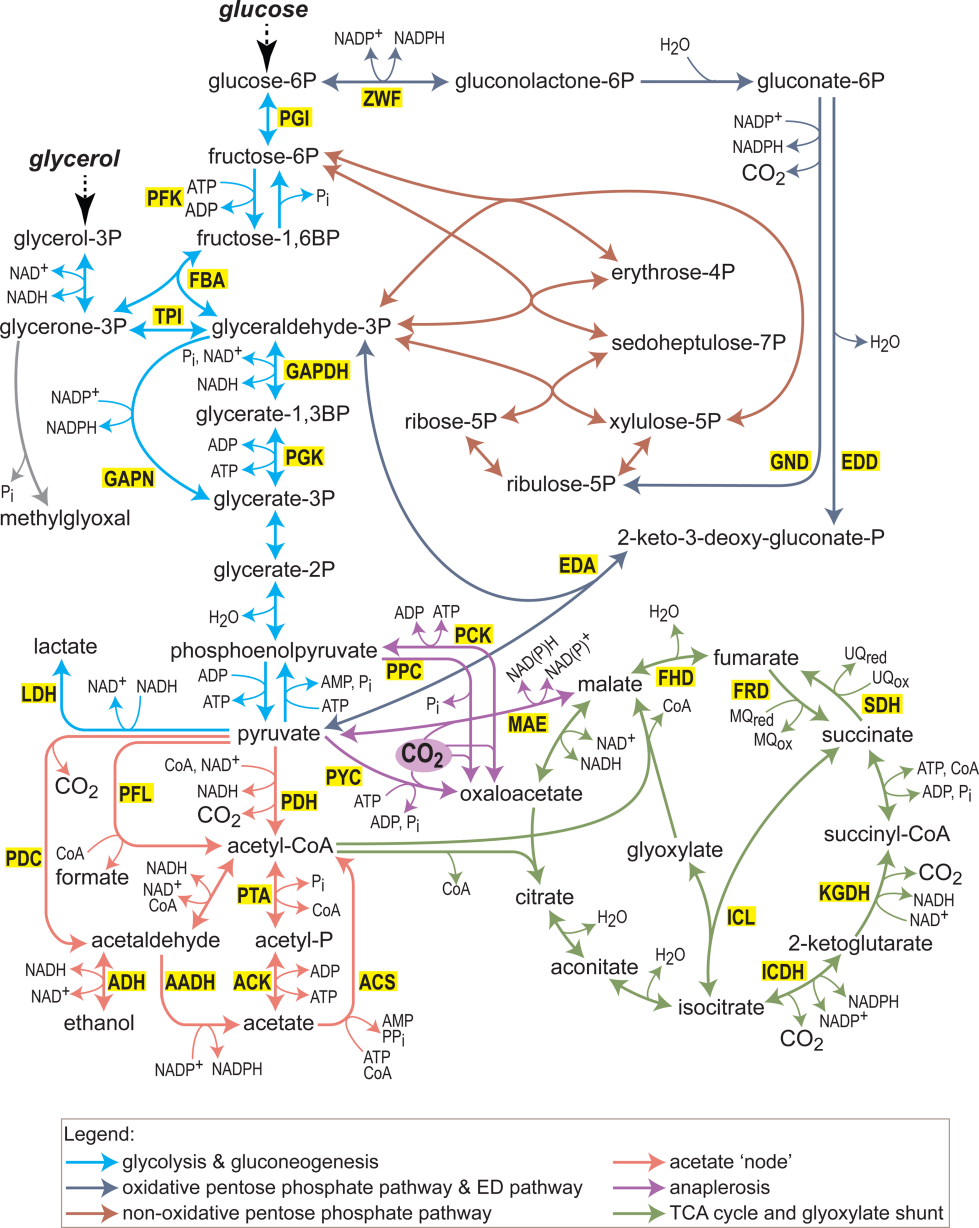
An overview of the structure of central metabolism in model organisms, such as *E. coli*, *C. glutamicum*, and *S. cerevisiae*, as discussed in the present paper. Each organism possesses only a subset of the enzymes shown in the figure. Compartmental separation (in case of eukaryotic organisms) is not shown. Glucose and glycerol are shown as representative carbon feedstocks. We have divided central metabolism into different generalized pathways, as indicated by the colors of the arrows. Enzymes mentioned in the text are shown with a yellow background. Some anaplerotic reactions use bicarbonate instead of CO_2_; for the sake of simplicity, we write CO_2_ as the substrate of all of them. Abbreviations: ACK, acetate kinase; ACS, acetyl-CoA synthetase; ADH, alcohol dehydrogenase; AADH, acetaldehyde dehydrogenase; EDA, 2-keto-3-deoxygluconate 6-phosphate aldolase; EDD, phosphogluconate dehydratase; FBA, fructose-bisphosphate aldolase; FHD, fumarate hydratase; FRT, fumarate reductase; GAPDH, glyceraldehyde 3-phosphate dehydrogenase (phosphorylating); GAPN, non-phosphorylating glyceraldehyde 3-phosphate dehydrogenase; GND, 6-phosphogluconate dehydrogenase (decarboxylating); ICDH, isocitrate dehydrogenase; ICL, isocitrate lyase; KGDH, 2-ketoglutarate dehydrogenase; LDH, lactate dehydrogenase; MAE, malic enzyme; PKF, 6-phosphofructokinase; PGI, glucose-6-phosphate isomerase; PGK, phosphoglycerate kinase; PCK, phosphoenolpyruvate carboxykinase; PDC, pyruvate decarboxylase; PDH, pyruvate dehydrogenase; PFL, pyruvate formate-lyase; PPC, phosphoenolpyruvate carboxylase; PTA, phosphate acetyltransferase; PYC, pyruvate carboxylase; SDH, succinate dehydrogenase; TPI, triose-phosphate isomerase; ZWF, NADP^+^-dependent glucose-6-phosphate dehydrogenase.

Figure 1 presents the canonical central metabolism, on which the networks of several model biotechnological organisms — mainly *Escherichia coli*, *Corynebacterium glutamicum*, and *Saccharomyces cerevisiae* — are overlaid (ignoring compartmental localization). Key pathways are marked by specific colors. Enzymes to which we directly refer below are marked with a yellow background. We divide this review into several sections, each discussing the modifications made to a different central pathway or process with the aim of enhancing a particular biosynthetic flux. As the topic is quite extensive, our review focuses on several primary examples which, we believe, demonstrate the key aspects.

## Abolishing endogenous fermentation products channels flux toward desired pathways

Different organisms, grown under different conditions (e.g. carbon sources and presence or absence of molecular oxygen), produce different combinations of compounds. While it is clear that fermentation, in the absence of electron acceptors, results in the conversion of most carbon input into fermentation products, it is important to emphasize that many organisms also produce large amounts of such products under aerobic conditions, e.g. acetate production by *E. coli*, ethanol by yeasts, and lactate by cancerous cells. This ‘overflow metabolism’ phenomenon is the center of a wide range of research efforts (e.g. [15–18]). For biotechnological applications, these endogenous fermentation products mostly represent a net loss of carbon which could otherwise be converted into a desired compound. It is, therefore, a common metabolic engineering practice to eliminate competing fermentation pathways with the aim of increasing the conversion yield of feedstock to product [19,20].

Multiple fermentation-related enzymes are targets for disruption: (i) lactate dehydrogenase (LDH), to eliminate lactate production; (ii) alcohol dehydrogenase, to eliminate ethanol biosynthesis; (iii) acetate kinase and/or phosphate acetyltransferase (PTA), to eliminate acetate production; (iv) pyruvate formate-lyase (PFL), to eliminate formate production; and (v) fumarate reductase (FRD), to eliminate succinate production. Deletion of the genes encoding these enzymes, or a subset of them, was shown to dramatically increase the production of hydrogen [21], pyruvate [22–24], lactate [25], succinate [26–28], malate [22,29], 2,3-butanediol [30–32], acetoin [31], acetyl-CoA [33], ethanol [34,35], *n*-butanol [36], branched-chain alcohols [37], tryptophan [38], citramalate [39], and many other chemicals of interest. Disruption of other enzymes, such as phosphoenolpyruvate (PEP) synthetase, PEP carboxylase (PPC), pyruvate kinase, pyruvate dehydrogenase (PDH), pyruvate oxidase, threonine decarboxylase, and 2-ketobutyrate formate-lyase, is less common, but was also shown to increase the yield of some products (e.g. [23,25,28,29]).

Unsurprisingly, disruption of endogenous fermentation enzymes commonly resulted in lower growth rate and redistribution of central metabolism flux to compensate for the disturbance of cellular ATP and NAD(P)H balance. For example, the simultaneous disruption of multiple fermentation-related enzymes in *E. coli* has not only led to the accumulation of pyruvate but also resulted in the increased activity of the alternative anaplerotic enzyme PEP carboxykinase (PCK, see section below), as well as an enhanced oxidative phosphorylation, which was required to balance the increased cellular NADH/NAD^+^ ratio [23]. In another study, deletion of the genes encoding for LDH and PFL in *E. coli* led to a high NADH/NAD^+^ ratio which inhibited dihydrolipoamide dehydrogenase, an essential component of the PDH complex. The authors were able to isolate a mutant strain in which a point mutation in this enzyme (E354K) substantially reduced its sensitivity to NADH and therefore enabled a high rate of pyruvate oxidation to acetyl-CoA [40]. In some cases, the disruption of fermentation enzymes resulted in higher biomass yield, and, surprisingly, a higher consumption rate of the sugar feedstock [30]. The latter finding may be explained by the fact that more glucose must now be consumed via alternative pathways with lower ATP yields to supply the energy once provided by the deleted fermentation routes.

In several studies, the ‘trimming’ of natural fermentation pathways was so intensive that the resulting strain became auxotrophic, as was shown in *E. coli*, where the disruption of PDH, PFL, and pyruvate oxidase not only enhanced pyruvate or lactate production but also rendered the cell dependent on external supply of acetate [24,25]. Such engineering is reasonable only if the externally supplied compound is considerably cheaper (and/or required in much smaller amounts) than the product. An advantage of this approach is that the concentration of the externally provided compound can control the growth of the organism, thereby enabling a tuning of cell growth *versus* product biosynthesis [25].

## Diverting flux toward NADPH production enhances NADPH-consuming pathways

The biosynthesis of many economically interesting products requires high investment of reducing power in the form of NADPH, which, in turn, necessitates increasing the regeneration rate of this essential cofactor [41]. NADP^+^ is endogenously reduced to NADPH via several routes and enzymes (which not all microbes share): glucose-6-phosphate dehydrogenase (ZWF, NADP^+^-dependent glucose-6-phosphate dehydrogenase) and 6-phosphogluconate dehydrogenase (GND, 6-phosphogluconate dehydrogenase, decarboxylating) of the oxidative pentose phosphate pathway, NADP-dependent malic enzyme (MAE) working in the decarboxylation direction, isocitrate dehydrogenase (ICDH) of the TCA (tricarboxylic acid) cycle, and the membrane, proton-translocating transhydrogenase (mTH) [42]. Increasing the metabolic flux through these enzymes, via the overexpression of their corresponding genes and deletion of competing pathways, was shown in many studies to enhance the NADPH-dependent biosynthesis of various products.

For example, blockage of normal glycolytic flux, via the disruption of glucose-6-phosphate isomerase (PGI) or 6-phosphofructokinase (PFK), channeled glucose toward the oxidative pentose phosphate pathway and/or the Entner–Doudoroff (ED) pathway (enzymes EDA, 2-keto-3-deoxygluconate 6-phosphate aldolase, and EDD, phosphogluconate dehydratase, in Figure 1). This resulted in increased regeneration of NADPH that supported enhanced production of various commodities, including hydrogen [43,44], lysine [45], valine [46], arginine [47], ornithine [48], lycopene [49], 2-chloropropionic acid [49], and terpenoids [50]. In some of these cases, rather than completely removing PGI, which often results in serious growth retardation, it was possible to reduce the expression level of its gene via replacement of its start codon ATG with GTG [47,48]. In other studies, it was shown that overexpression of the genes of the oxidative pentose phosphate pathway (e.g. ZWF) is enough to channel significant flux toward NADPH regeneration [51–53]. Moreover, as ZWF and GND tend to be inhibited by NADPH, their replacement with NADPH-insensitive counterparts can support higher flux via the pathway [44].

Another study took the idea of diverting flux toward NADPH regeneration to the extreme: glycolytic flux in *C. glutamicum* was completely blocked by deleting the genes of both PFK and glyceraldehyde 3-phosphate dehydrogenase (GAPDH), such that (almost) all glucose molecules were completely oxidized to CO_2_. This provided nearly stoichiometric amounts of NADPH via a cyclic activity of the oxidative pentose phosphate pathway [54]. (In reality, one-fourth of the glucose was converted into glycerol, thereby reducing NADPH yield from 12 — maximal stoichiometric yield — to 9.) This enabled the resting cells to serve as a highly efficient catalysts for the NADPH-dependent reduction of the methyl acetoacetate to (*R*)-methyl-3-hydroxybutyrate [54].

In *E. coli* growing on glucose under standard aerobic batch conditions, 35–45% of NADPH is produced via the mTH [42], which prompted many metabolic engineers to overexpress the corresponding gene in order to increase NADPH levels. For example, such overexpression resulted in enhanced NADPH-dependent production of poly(3-hydroxybutyrate) (PHB) in *E. coli* [55]. Similarly, the introduction of *E. coli* mTH to *C. glutamicum* improved lysine bioproduction [56]. Overexpression of NAD kinase, which increases the concentration of the overall NADP(H) pool, was further shown to enhance NADPH-dependent bioproduction routes [57–59].

Less ubiquitous enzymes can also channel flux toward NADPH regeneration. The most interesting of these is the NADP-dependent non-phosphorylating glyceraldehyde 3-phosphate dehydrogenase. Overexpression of the *Clostridium acetobutylicum* gene encoding this irreversible enzyme — directly oxidizing glyceraldehyde 3-phosphate to 3-phosphoglycerate [60] — was shown to enhance the NADPH-dependent production of compounds such as lysine [61] and PHB [62]. Finally, moving away from central metabolism, NADPH availability could be increased by providing reduced compounds that can donate their electrons to NADP, such as formate [63] or phosphite [64].

## Alternative glycolytic architectures provide pathway precursors

**Figure 2.**
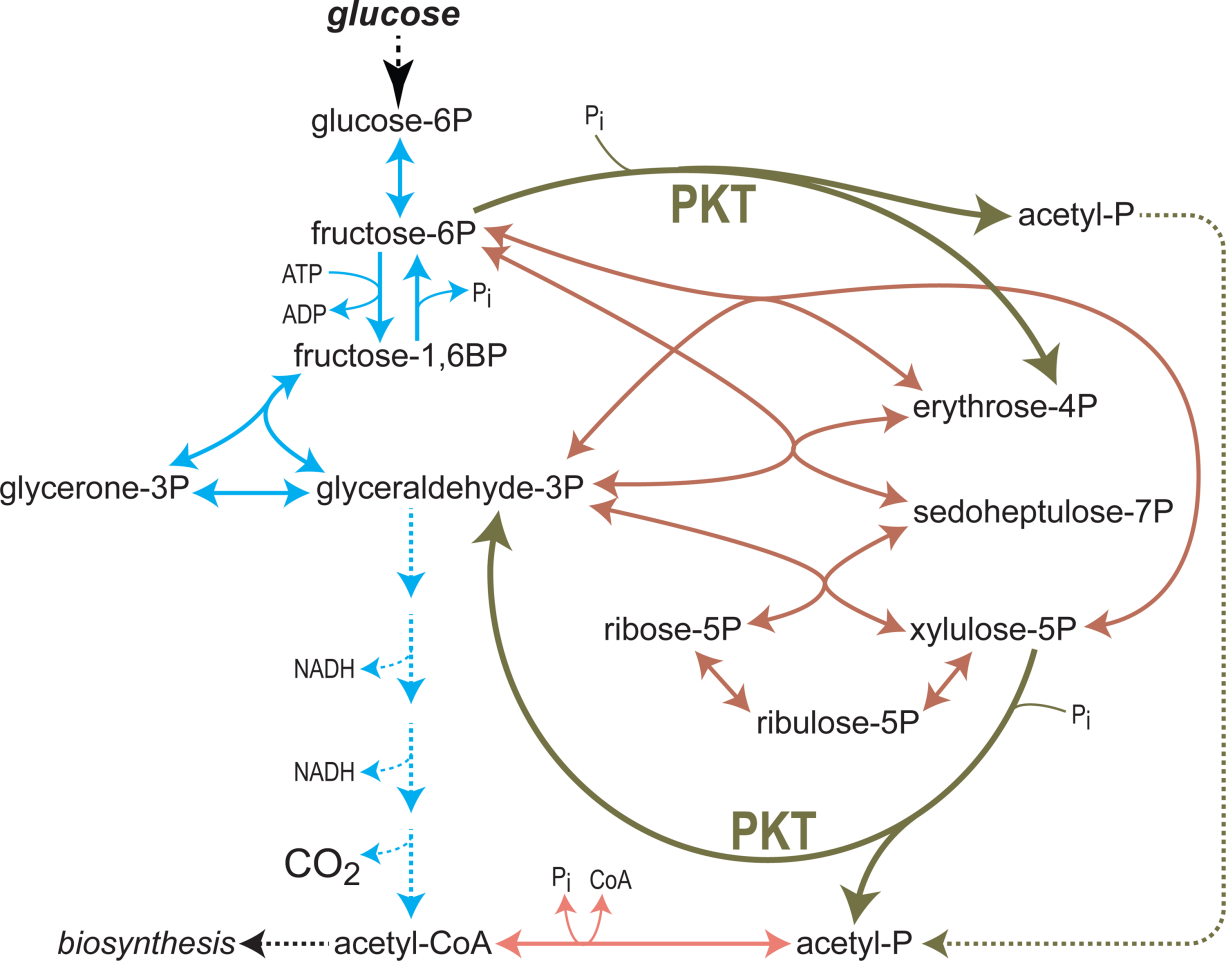
PKT enables direct conversion of sugar phosphates to acetyl phosphate and thus acetyl-CoA. The two reactions catalyzed by the PKT are shown in dark green. Note that the conversion of sugars to acetyl-CoA via PKT is carbon- and redox-neutral, while glycolysis emits CO_2_ and generates two NADH molecules per each acetyl-CoA produced.

Apart from diverting flux toward increased NADPH production, glycolytic bypasses can also serve to channel carbon toward key precursors of bioproduction pathways, thus supporting their enhanced activity. Perhaps, the most famous example is the use of the enzyme phosphoketolase (PKT) to bypass most of central metabolism, generating activated acetyl moieties directly from phosphosugars [65]. PKT combines ketol cleavage, dehydration, and phosphorolysis to cleave xylulose 5-phosphate to glyceraldehyde 3-phosphate and acetyl phosphate, and fructose 6-phosphate to erythrose 4-phosphate and acetyl phosphate (Figure 2) [66]. Acetyl phosphate can, then, be converted into the central metabolite acetyl-CoA.

The use of PKT offers two main advantages for the biosynthesis of commodities for which acetyl-CoA serves as a precursor: (i) a direct route from upstream metabolism, i.e. phosphosugars, to acetyl-CoA, which forces high flux toward this central metabolite and (ii) conversion of phosphosugars into acetyl-CoA without the loss of carbon from pyruvate decarboxylation [67], which could potentially increase product yield. In theory, the PKT route could serve to convert glucose into three acetyl-CoA molecules, instead of two via natural glycolysis. However, unlike the endogenous glycolytic pathway, no reducing power is gained via the PKT route, which limits any downstream pathway that heavily relies on NAD(P)H consumption. For example, the biosynthesis of fatty acids requires two NAD(P)H molecules per one acetyl-CoA and therefore optimally operates with endogenous glycolysis that provides this exact ratio.

One of the first uses of PKT in metabolic engineering was to enhance ethanol production from xylose in *S. cerevisiae* [68]. The enzyme was further harnessed to support increased bioproduction of other acetyl-CoA-derived compounds, such as *n*-butanol [69], succinate [70], glutamate [71], isoprenoid [72], polyhydroxybutyrate [73], fatty acid ethyl esters [74], and 6-methylsalicylic acid [75]. Yet, the biosynthesis of these compounds also requires reducing power, hence limiting the contribution of the NAD(P)-free PKT route. On the other hand, as the conversion of acetyl-CoA into acetate [67,76] or acetone [77,78] is redox-free, the production of these chemicals can benefit the most from the PKT glycolytic bypass. One especially interesting idea is the use of PKT in cyanobacteria for increased acetone biosynthesis: the CO_2_ fixed into sugar phosphates was directly channeled to acetyl-CoA and acetone production without any loss of carbon [78].

**Figure 3.**
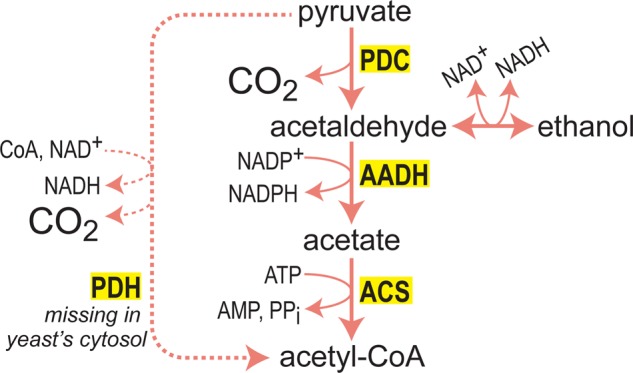
A synthetic ‘pyruvate dehydrogenase shunt’ engineered in yeast for increased acetyl-CoA production. Overexpression of AADH and ACS, in a background of high pyruvate decarboxylase activity, results in a short pathway that converts pyruvate into acetyl-CoA, as is the case for the PDH complex. The PDH shunt, however, dissipates considerably more energy by hydrolyzing ATP.

Increasing the availability of acetyl-CoA can be done by other alternative glycolytic structures. For example, *S. cerevisiae* does not have a cytosolic pyruvate dehydrogenase, as is the case in *E. coli*. To increase acetyl-CoA production, the genes encoding acetaldehyde dehydrogenase and acetyl-CoA synthetase (ACS) were overexpressed. Together with the highly active pyruvate decarboxylase (endogenous), these enzymes constituted a ‘pyruvate dehydrogenase’ shunt that efficiently converts pyruvate into acetyl-CoA (Figure 3) [79]. Similarly, establishing high activity of ACS in the cytosol of the yeast *Yarrowia lipolytica* was shown to divert acetaldehyde and acetate (produced from pyruvate decarboxylase) toward acetyl-CoA, thereby supporting the biosynthesis of the downstream metabolite 2-ketoglutarate [80]. Interestingly, the authors also established high activity of ATP-citrate lyase in the yeast cytosol, regenerating acetyl-CoA from citrate, which was originally produced in the mitochondria and then transported to the cytosol [80].

Activation of the ED pathway was found to support increased pyruvate and acetyl-CoA availability and PHB biosynthesis in *E. coli* [81]. This is likely because the ED pathway can sustain higher glycolytic flux, per amount of overall enzyme, when compared with the common (Embden–Meyerhof pathway, EMP) glycolysis [82]. Activation of serine degradation, providing yet another route for pyruvate production, further enhanced accumulation of PHB [81], demonstrating that multiple glycolytic bypasses can operate in parallel in a beneficial manner. The ED pathway was further shown to increase isoprenoid biosynthesis via the 2-C-methyl-d-erythritol 4-phosphate pathway (MEP), as it provides balanced amounts of glyceraldehyde 3-phosphate and pyruvate, both of which serve as the direct precursors of this metabolic route [83,84].

In many key biotechnological organisms, such as *E. coli* and *C. glutamicum*, the phosphotransferase system (PTS) serves as a key cellular entry point of sugars (e.g. [85,86]). The PTS transfers an activated phosphate group from PEP to the sugar feedstock — e.g. glucose and fructose — thus activating it for use in central metabolism. This forces half of the PEP molecules to be converted into pyruvate, instead of being used for other biosynthetic purposes, for example, anaplerosis and production of aromatic amino acids. (We further note that while *C. glutamicum* and *S. cerevisiae* possess an anaplerotic pyruvate carboxylase, PYC, *E. coli* does not have this enzyme and therefore solely relies on PEP carboxylation for anaplerosis.) Hence, a useful alteration of sugar fermentation results from the replacement of the PTS with a sugar-kinase enzyme that does not consume PEP [87,88], thus freeing this central intermediate to support other metabolic processes. For example, replacement of PTS by ATP-dependent phosphorylation increased the biosynthesis of shikimic acid — a PEP-derived intermediate of aromatic amino acid biosynthesis — in *E. coli* [89], and enhanced lysine yield in *C. glutamicum* [90].

Disruption of the endogenous TPI (triose-phosphate isomerase) is a powerful tool to manipulate sugar fermentation, such that only half of its flux proceeds via the normal glycolysis, while the other half (starting from dihydroxyacetone phosphate, DHAP) is channeled toward the biosynthesis of products of interest [91]. This approach was used, for example, to divert half of the glycolytic flux toward methylglyoxal and downstream production of 1,2-propanediol and 1-propanol [92]. Similarly, disruption of TPI resulted in high metabolic conversion of DHAP into glycerol and further into 1,3-propanediol [93].

In some cases, native glycolysis was not disrupted but rather enhanced by an elevated level of its enzymes, in an attempt to increase its flux and support the bioproduction of downstream compounds.^1^ For example, overexpression of the native components of the PDH complex (i.e. *aceEF* and *lpd*), as well as other glycolytic enzymes, e.g. GAPDH and phosphoglycerate kinase (PGK), was used to increase acetyl-CoA availability for the downstream production of butanol in *E. coli* [94,95]. In a similar manner, elevated levels of the native GAPDH and PGK increased the carbon flux toward malonyl-CoA, a direct product of acetyl-CoA carboxylation [96]. Overexpression of the endogenous genes encoding for TPI and fructose-bisphosphate aldolase led to increased production of acetyl-CoA and its downstream product PHB [97]. Finally, the biosynthesis of glycolytic products can also be enhanced by increasing the availability of essential cofactors, e.g. supplementation of pantothenic acid, a CoA-precursor, resulted in increased levels of CoA and acetyl-CoA and enhanced the production of downstream products, such as isoamyl acetate [98].

We conclude this section by noting that instead of replacing (EMP) glycolysis, it could be beneficial to introduce this pathway into organisms that do not possess it, as the ATP yield of the pathway is higher than the alternatives. For example, *Pseudomonas putida* metabolizes glucose through a cycle formed by enzymes of the ED, EMP, and pentose phosphate pathways, but cannot operate a linear glycolytic flux as it lacks PFK [99]. Introducing the enzymes of the EMP pathway to this organism enabled linear flux, increasing PHB production 3-fold, presumably due to increased ATP availability [100].

## Alternative anaplerosis increases ATP availability and product yield

**Figure 4.**
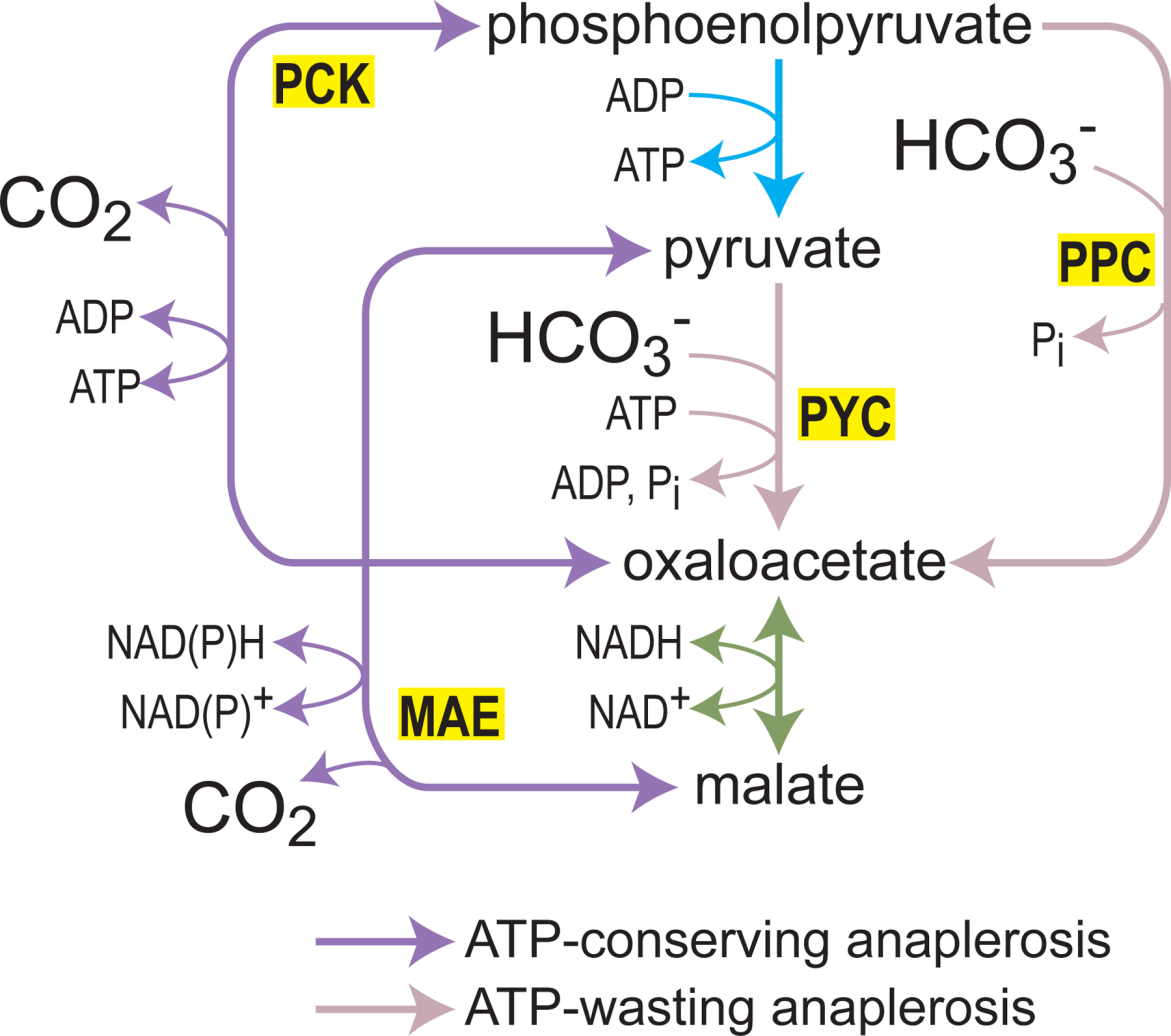
An overview of different anaplerotic reactions and their ATP economy. PPC and PYK are very efficient carboxylating enzymes, but dissipate an ATP equivalent. PCK and the MAE can perform anaplerosis only at high CO_2_ and at low rate, but can support the conservation of an ATP equivalent, contributing to high cellular energetic state.

Anaplerotic reactions convert glycolytic intermediates into those of the TCA cycle, thus enabling the net biosynthesis of di- and tricarboxylic acids. Some of these compounds, such as succinate, malate, and citrate, are economically interesting by themselves, while others are precursors for the biosynthesis of chemicals of interest, e.g. aconitate which can be decarboxylated into itaconate, a top value-added chemical [101]. In most organisms, anaplerotic reactions are carried either by PPC (e.g. in *E. coli*) or by PYC (e.g. *S. cerevisiae*). These can be replaced by enzymes that usually support the reverse, decarboxylation direction, i.e. PCK or MAE. Such a replacement results in the net gain of an ATP molecule (Figure 4): while PPC releases the activated phosphate of PEP without generating ATP and PYC consumes the ATP produced by pyruvate kinase, PCK generates ATP from PEP during its carboxylation, and the malic enzyme carboxylates pyruvate without consuming ATP.

The potential to gain an additional ATP by the use of PCK or MAE has led several groups to replace PPC or PYC with these enzymes. It was expected that the increased ATP production would reduce the fraction of carbon channeled toward ATP supply (e.g. oxidation to provide reducing power for oxidative phosphorylation and acetate production) and increase the carbon flux toward the desired product. Indeed, replacement of PPC with PCK in *E. coli* approximately doubled the intracellular ATP concentration [102,103]. As expected by the increased ATP availability, production of succinate increased by up to 7-fold by such engineering, but only when bicarbonate was added [104,105]. Notably, replacing PPC with PCK resulted in a marked decrease in the growth rate of the engineered *E. coli* strains [102,103]. Likewise, overexpression of PCK in a PYC-disrupted *S. cerevisiae* strain sustained growth only at elevated CO_2_ concentration and only at half the normal growth rate [106]. It is interesting to note that a long-term cultivation of a succinate-producer *E. coli* strain resulted in increased activity of PCK, which has become the major anaplerotic enzyme, vividly demonstrating the advantage of increasing ATP yield during the production of a TCA cycle intermediate [107].

Replacement of PYC or PPC with MAE has led to similar results. For example, increasing the expression of NADP-dependent MAE in *E. coli* enhanced the production of succinate and other C4 metabolites but only at increased inorganic carbon concentration [108,109]. An NAD-dependent MAE from *E. coli* was able to rescue the growth of a PYC-disrupted *S. cerevisiae* under high CO_2_ concentration, but only after the emergence of a point mutation that switched the cofactor preference of MAE to NADPH [110].

The low growth rate of the PCK- and the MAE-dependent strains, as well as their dependence on high concentrations of inorganic carbon and NADPH (in the case of the MAE), points to a general phenomenon: a trade-off between efficiency and rate. Specifically, unlike PPC and PYC, which prefer the carboxylation direction (Δ_r_G′*^m^* approximately −40 kJ/mol and approximately −14 kJ/mol, respectively, at pH 7 and ionic strength of 0.25 mM [111]), where Δ_r_G′*^m^* corresponds to the reaction change in Gibbs energy under metabolite concentration of 1 mM [112]), PCK and MAE prefer the decarboxylation direction (Δ_r_G′*^m^* approximately −6 kJ/mol and approximately −4 kJ/mol, respectively). Therefore, operating at high concentration of inorganic carbon and with the reduced NADP pool (rather than the oxidized NAD pool [113]) is essential to provide sufficient driving force to push PCK and MAE toward carboxylation. Even then, the relatively low driving force [114,115] and poor kinetics of these enzymes in the carboxylation direction — low affinity toward inorganic carbon and low *k*_cat_ — limit the anaplerotic flux and constrain the growth of the engineered strains. Hence, metabolic engineers trying to maximize C4 production are faced with a dilemma: should they increase product yield at the expense of biosynthesis rate (PCK/MAE) or should they favor high flux even if a considerable fraction of the carbon feedstock is wasted to energize the cell (PPC/PYC)?

## Disruptions of the TCA cycle direct flux toward metabolites of interest

The enzymes of the TCA cycle are also a common target of disruption for many purposes. A common gene deletion within the TCA cycle, as discussed in the first section, is that of FRD, typically used to abolish the unwanted production of succinate. Yet, it is important to note that this deletion is only partially useful, as succinate dehydrogenase (SDH, preferentially using ubiquinone instead of menaquinone, as is the case for FRD) can also operate in the reductive direction [116]. Another common disruption is that of 2-ketoglutarate dehydrogenase (KGDH), in order to avoid carbon loss via the oxidative cyclic flux and further limit the consumption of acetyl-CoA, thus freeing this key precursor to be used in bioproduction pathways (e.g. [117,118]). Notably, the disruption of KGDH does not interfere with the production of essential cellular building blocks, e.g. oxaloacetate, 2-ketoglutarate, and succinyl-CoA, as they can be produced via the independent operation of the two branches of the TCA cycle, that is, reductive flux from pyruvate/PEP to oxaloacetate and succinyl-CoA and oxidative flux from citrate to 2-ketoglutarate.

As is the case in glycolysis, it was shown that enhancing the flux via the native TCA cycle could serve to support increased bioproduction of its intermediates. For example, succinate biosynthesis was shown to increase in *E. coli* by overexpression of FRD — supporting higher flux via the reductive arm of the cycle [119–121]. Production of succinate was also shown to benefit from the disruption of KGDH and redirection of TCA flux toward the glyoxylate shunt. For example, disruption of KGDH in *S. cerevisiae* resulted in isocitrate cleavage to succinate, which, together with the deletion of the SDH gene to prevent recycling of succinate, led to a 4.8-fold higher titer of this dicarboxylic acid [122]. Disruption of SDH and activation of the glyoxylate shunt, via the deletion of its repressor (IclR) and/or deletion of the KGDH gene, were also shown to substantially increase succinate production in *E. coli* [123–126]. Similarly, increased fumarate production was observed upon deletion of the *iclR* gene and disruption of all fumarate hydratase isozymes [127].

**Figure 5.**
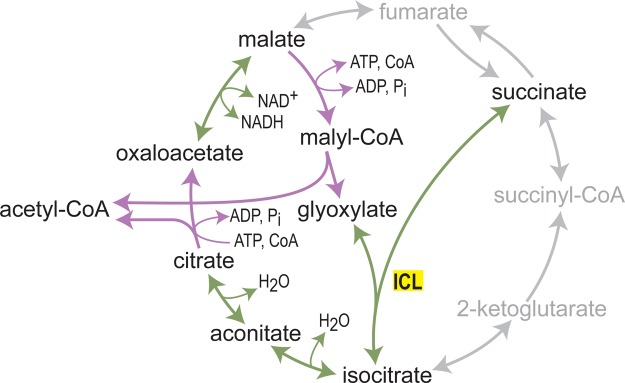
A reverse glyoxylate shunt, metabolizing succinate into two acetyl-CoA molecules. Reactions specific to this reverse activity are shown in purple: malyl-CoA synthetase and lyase, and ATP-citrate synthase.

A disruption of citrate synthase resulted in a 2-ketoglutarate auxotrophic strain (required for the biosynthesis of glutamate, glutamine, proline, and arginine), whose prototrophic growth could be reinstituted by the reverse activity of isocitrate lyase in the presence of succinate and glyoxylate [128]. The authors of this study have further demonstrated that glyoxylate can be produced *in situ* via the combined activity of heterologously expressed malyl-CoA synthetase and lyase, and that a foreign ATP-citrate lyase can serve as the sole source of oxaloacetate (for the biosynthesis of aspartate, asparagine, lysine, methionine, threonine, and isoleucine). Together, this work serves as a preliminary step for the establishment of a reverse glyoxylate shunt, that is, cleavage of succinate into two acetyl-CoA moieties (Figure 5) [128,129].

**Figure 6.**
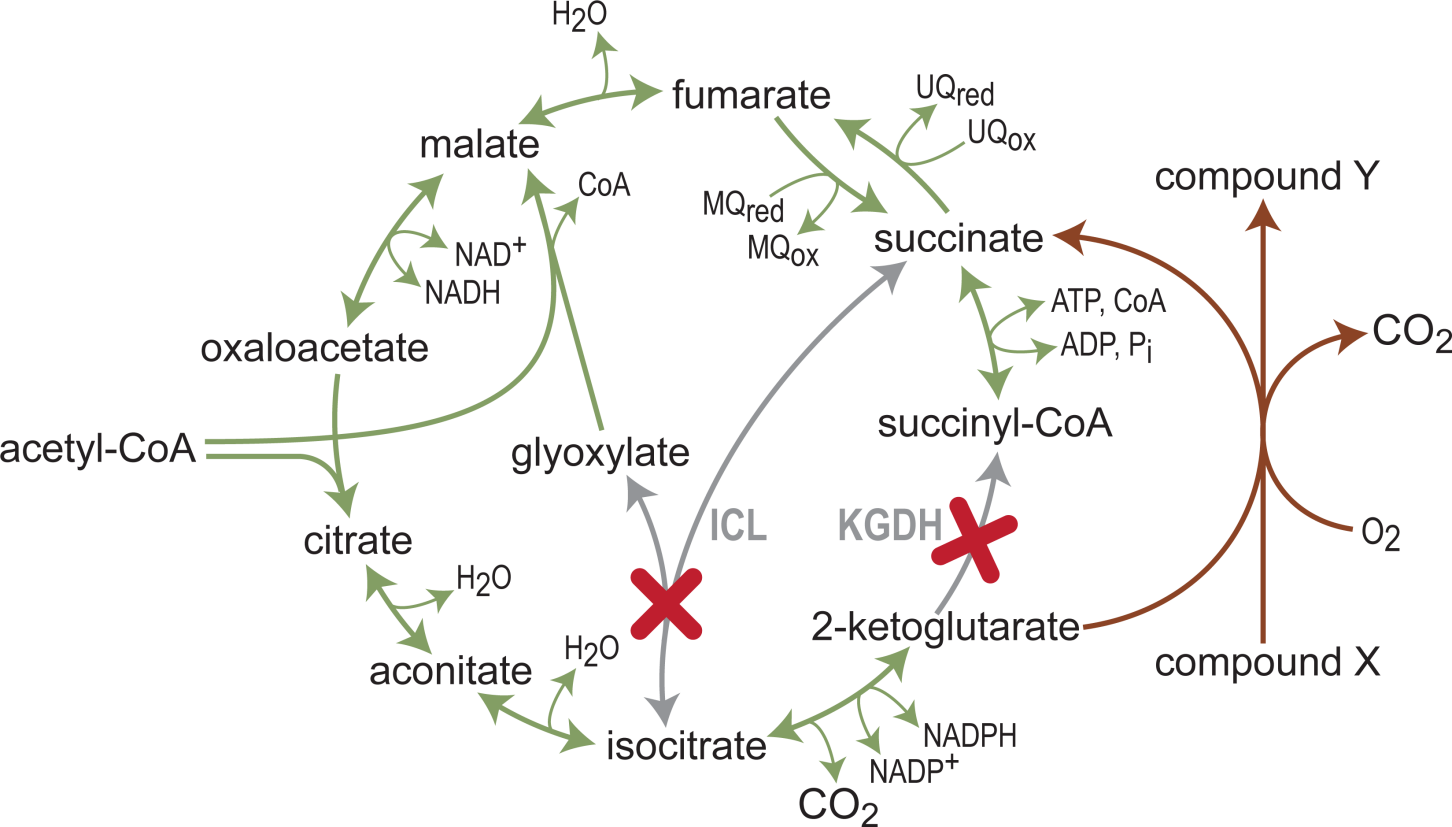
Coupling cellular growth to a desired bioconversion. A strain disrupted in isocitrate lyase (ICL) and KGDH cannot grow on a minimal medium as it is blocked in all routes for citrate/isocitrate/2-ketoglutarate recycling. Overexpression of a 2-ketoglutarate-dependent dioxygenase (brown line) provides a sink for 2-ketoglutarate and thus enables growth, while catalyzing the conversion of a feedstock compound X to a desired compound Y.

An interesting rewiring of the TCA cycle was established by disrupting both KGDH and ICDH. This strain was not able to grow on a minimal medium with glucose or glycerol as carbon sources, as the downstream flux from citrate was completely blocked [130]. Growth of this strain was restored only when an alternative sink for 2-ketoglutarate was established via a 2-ketoglutarate-dependent dioxygenase (Figure 6) [131], therefore enabling the coupling of cellular growth to the biosynthesis of 4-hydroxyisoleucine (isoleucine + 2-ketoglutarate + O_2_ → 4-hydroxyisoleucine + succinate + CO_2_) [130] or 4-hydroxyproline (proline + 2-ketoglutarate + O_2_ → 4-hydroxyproline + succinate + CO_2_) [132]. In both these studies, high conversion yields of substrate to product were achieved, vividly demonstrating a central lesson in holistic bioengineering: if it is possible to couple the biosynthesis of a target product to cellular growth, the cell will naturally adapt its metabolism toward increased biosynthetic rate.

## Concluding remarks

This review explores and exemplifies the importance of considering the entire metabolic network, and especially central metabolism, when optimizing the biosynthesis of a product of interest. Through the examples presented in this review, several general principles of holistic bioengineering emerge. First and foremost, regardless of the exact pathway or process in question, the manipulation of cellular metabolism is mostly aimed at one of three key goals: (i) deleting endogenous pathways that compete with the biosynthesis of a desired product. These include fermentation pathways in which carbons leak out of the cell, oxidative pathways — such as the TCA cycle — that release carbon as CO_2_, or simply metabolic highways that channel flux away from the required biosynthetic route. (ii) Increasing flux toward a key precursor of the biosynthesis route. This can be performed by the overexpression of endogenous enzymes or the introduction of foreign enzymes and pathways that directly convert feedstock to precursor. The most commonly pursued biosynthetic precursor is acetyl-CoA; numerous studies have developed innovative strategies to increase the level of this central metabolite toward enhancing the production of a myriad of downstream products. Other metabolic precursors, such as pyruvate or 2-ketoglutarate, have received less attention; however, considering their importance for the biosynthesis of multiple chemicals, we can expect more efforts to be directed toward their enhanced synthesis. (iii) Manipulating the energy and redox state of the cell to rewire cellular fluxes toward bioproduction. Here, we differentiate between two complementary approaches. A general strategy is to increase the ATP levels in the cell — e.g. via ATP-generating anaplerosis or more efficient glycolysis — such that less of the carbon feedstock is directed toward energy production and more toward biosynthesis. A more focused approach is to increase the availability of an energy carrier that is required in high amounts for a specific biosynthetic process. The most common example is NADPH, a high supply of which is essential for the production of numerous chemicals.

Another important principle is the inherent trade-off between rate and yield, especially from a thermodynamic point of view. That is, ATP-efficient pathways tend to operate under a low thermodynamic driving force. This leads to one of two outcomes: low pathway rate or high investment in pathway enzymes, leading to protein burden [114]. One of the most illuminating examples of this phenomenon is the prevalence of the ED pathway. While this pathway generates only half the ATP molecules the EMP glycolysis does, its higher thermodynamic driving force enables high flux at low protein investment and hence, it is the preferred pathway in many microorganisms that can produce ATP via means other than glycolysis, e.g. respiration or photosynthesis [82].

An additional fundamental lesson relates to harnessing natural selection for increased bioproduction. In most cases, sustaining high flux via a biosynthetic route does not benefit the microbe, but rather consumes resources that could otherwise be used to sustain growth, i.e. the activity of the pathway has a negative impact on fitness. As such, strain instability, where short-term cultivation results in the rise of mutations that abolish the activity of a biosynthetic route, is a common hurdle that limits many bioproduction projects (e.g. [133,134]). Yet, in some cases, it is possible to engineer the bioproduction strain such that the activity of the biosynthetic route will be beneficial, or even essential for microbial growth. As growth is coupled to pathway activity, strain evolution is highly unlikely to disrupt it. As discussed above, a common example for this is the deletion of competing fermentation pathways, such that ATP-producing, redox-balanced fermentation can proceed only via a specific pathway leading to a desired product. Another interesting example, discussed in the TCA cycle section, is the use of 2-ketoglutarate-dependent dioxygenase as sole way to recycle 2-ketoglutarate, thus coupling growth to a required biocatalysis. Harnessing natural selection in such a way can serve more than to just ensure the stability of a biosynthetic route: it can also be used to enhance its activity. While most metabolic engineering efforts require multiple design-test cycles — tweaking gene expression levels and monitoring the effect on production — these can be avoided if direct selection for high pathway activity is possible, thus saving time and labor.

In most cases, the rewiring of cellular metabolism was rationally designed based on the knowledge and experience of the authors. In other cases, a computational strategy was taken to systematically explore all possible alternations of central metabolism that could lead to beneficial results (e.g. [135–141]). While both approaches are valid, it is fair to say that in the long run, precise fine-tuning of the endogenous cellular metabolism will vastly benefit from specialized software and computational tools. Yet, for such tools to truly reinvent the field of metabolic engineering, they should involve much more than a stoichiometric analysis, as is commonly the case now. Instead, thermodynamics and kinetics of reactions should be considered [142–145], alongside known regulatory effects, such as allosteric inhibition or activation, as well as measured ranges of enzyme and metabolite concentrations (e.g. [113]). While obtaining sufficient data on these parameters is a not an easy task, the availability of -omics tools and databases can considerably assist in addressing this challenge.

We emphasize that while we focused on central metabolism in this review, a truly holistic bioengineering encompasses other aspects of cellular physiology, including amino acid, nucleotide and fatty acid metabolism, assimilation of inorganic elements such as nitrogen and sulfur, passive and active cellular transport, as well as transcription and translation. Each of these cellular processes can be — and should be — modified or rewired as to support the most efficient conversion of feedstock into product. We would like to finish by noting that even if we keep focusing on central metabolism, the challenge of wholly redrawing it — as opposed to mildly rewiring it, as is commonly done — is still open. To what extent can we reinvent key metabolic pathways? Can such dramatic changes be useful for biotechnological applications? Some pioneering studies, e.g. engineering an active Calvin Cycle in *E. coli* [146] — may start providing answers to these questions soon.

## Footnotes

1 We note that overexpression of an enzyme does not necessarily lead to an increased pathway flux. The effect of such overexpression will be dependent on the control the enzyme exerts on pathway activity, which is beyond the scope of this review.

## Abbreviations

ACS: acetyl-CoA synthetase
*C. glutamicum*: *Corynebacterium glutamicum*
DHAP: dihydroxyacetone phosphate
*E. coli*: *Escherichia coli*
ED: Entner–Doudoroff
EMP: Embden–Meyerhof–Parnas
GAPDH: glyceraldehyde 3-phosphate dehydrogenase (phosphorylating)
GND: 6-phosphogluconate dehydrogenase (decarboxylating)
ICDH: isocitrate dehydrogenase
KGDH: 2-ketoglutarate dehydrogenase
LDH: lactate dehydrogenase
MAE: malic enzyme
MEP: 2-C-methyl-d-erythritol 4-phosphate pathway
mTH: membrane, proton-translocating transhydrogenase
PFK: 6-phosphofructokinase
PGI: glucose-6-phosphate isomerase
PGK: phosphoglycerate kinase
PCK: phosphoenolpyruvate carboxykinase
PDH: pyruvate dehydrogenase
PEP: phosphoenolpyruvate
PFL: pyruvate formate-lyase
PHB: poly(3-hydroxybutyrate)
PTS: phosphotransferase system
PKT: phosphoketolase
PPC: phosphoenolpyruvate carboxylase
PYC: pyruvate carboxylase
*S. cerevisiae*: *Saccharomyces cerevisiae*
SDH: succinate dehydrogenase
TCA: tricarboxylic acid
TPI: triose-phosphate isomerase
ZWF: NADP^+^-dependent glucose-6-phosphate dehydrogenase
